# Debi Ghate and Richard E. Ralston: Why businessmen need philosophy: the capitalist’s guide to the ideas behind Ayn Rand’s *Atlas Shrugged*: New American Library (Penguin Group), New York, 2011, 316 pp, ISBN: 978-0-451-23269-4

**DOI:** 10.1007/s10202-012-0106-5

**Published:** 2012-04-26

**Authors:** Mario Garitta

**Affiliations:** Farmingville, NY USA

## Abstract

The essays in this book are meant to serve as an introduction to those ideas of Ayn Rand, which are of particular relevance to business people. Rand was known as a spirited defender of the *laissez*-*faire* free enterprise system. It is less commonly known that Rand was also deeply committed to the centrality of the enterprise of philosophy for both public and private life. The essays in this book try to bridge the gap between these two aspects of Rand’s thought. The results of the review of the book are mostly positive. The review attempts to separate the different themes in the book such as the importance of philosophy in general, the importance of philosophy for business, the philosophical defense of the free enterprise system and then to evaluate the evidence and arguments presented by the essayists for each claim.

The growing field of business ethics testifies to the importance of the discipline of philosophy for practical life. After decades of hearing rumors of the ‘death of philosophy’, suddenly philosophy has a vibrant role to play in the eminently practical endeavor of business. The same paradigm has been reenacted in medical and environmental ethics. It is significant that the important role which philosophy is now occupying in various practical endeavors did not emerge from within the self-reflection of modern philosophical reason. Whether we turn to Marxism, existentialism, analytical philosophy, deconstruction, phenomenology, or pragmatism, the result has been a diminished role for philosophy. Nor is this state of affairs accidental. In each of the above philosophy, an abstract enterprise is conceived with little relation to even one which stands in direct opposition to the realm of the practical. If we take business ethics as an example, this field developed out of aspects of practical life such as the environmental and consumer protection movement. In short, philosophers were in effect caught by surprise as questions that were obviously philosophical—questions concerning the nature and limits of corporate responsibility and the role of business in relation to the protection of the environment were all too naturally thrust in their direction. The failure or rather the methodological inability on the part of contemporary thinkers to anticipate the development of whole fields of endeavor is symptomatic of something amiss in the understanding of the relation between theory and practice expressed and embodied in modern philosophy. One would fully expect theory to provide a coherent basis for practice, and indeed this has been the case in the dominant traditions in the West. When the two can as a matter of principle no longer be correlated, when this fact is all but worn as a badge of honor, something is profoundly disordered in our understanding of either theory, practice or both. Needless to say, professional philosophers have indeed responded to the challenge of questions that have been foisted upon them, but they have done so in effect by thinking in spite of or outside virtually every major tradition of modern philosophy from analytical philosophy to Derrida, once again a symptom of a profound disorder within the house of modern philosophy itself.

If there is one modern philosophical movement in which the role of philosophy has never been doubted or diminished, it is in the ‘objectivist’ philosophy of Ayn Rand. Swimming against the current of virtually every major system of modern thought, Rand has consistently maintained the inexorability of philosophical reason for every aspect of human life, public as well as private. In Rand’s universe, it is ideas not material life conditions, or unconscious forces that for better or for worse shape the destiny of individuals and of nations.

Her spirited defense of capitalism as well as her critique of altruism and collectivism are solidly grounded in the objective realism of Aristotle. In Rand, one finds a coherent philosophical system with the central role occupied by metaphysics followed by epistemology, ethics and political philosophy. Accordingly to Rand, human beings as rational animals must inevitably confront questions concerning the nature of what is real, the purpose of human life and the character of knowledge, and hence, a broad philosophical framework which addresses these questions is unavoidable.

In their compendium *Why businessmen need philosophy,* editors Debi Ghate and Richard E. Ralston have assembled an impressive collection of essays that specifically addresses the issue of the philosophical grounding of business. Harry Binswanger’s essay “Philosophy: The Ultimate C.E.O” establishes a solid orientation for the theme of the book. Philosophy, Binswanger argues is indispensable for business because it is indispensable for human beings who strive to actualize their highest potential which is reason. Just as the C.E.O. of a corporation provides the broad-based conceptual framework within which such things as strategic goals and initiatives are possible, so is the job of philosophy to establish a comprehensive and coherent perspective on reality as a whole. Even the most practical and ‘down to earth’ choices concerning career pathways and financial issues impinge on epistemological, ethical and even metaphysical questions. Failure to explicitly engage the large issues of philosophy is to decide them by default, thus philosophical choices are unavoidable. Binswanger does not shrink from the implications of this view. The necessity of illuminating human actions in terms of the broad frameworks which only philosophy can provide implies that epistemological, ethical and ontological frameworks are not only always operative, but guide, direct and even determine the character of our actions. Thus, it is ideas that shape the destiny of both individuals and of nations.

In our postmodern era, we are all too likely to regard such broad-based claims as naïve. Are not philosophical concepts a mere epiphenomenon of material life conditions, economic forces, instinctual drives and the ‘will to power’? Don’t philosophical concepts themselves deconstruct once examined? If this postmodernist view of the diminished role of philosophy is to prevail with integrity, it must come to terms with some of the very compelling counterexamples cited by Binswanger. The semi-feudal society of Russia went Communist. The rich capitalist societies of Britain and Sweden turned socialist. America in 1776 then poorer than Russia was in 1917 turned capitalist. Each of these cases can in defiance of economic realities be traced to the influence of powerful philosophical concepts (e.g., the influence of John Locke upon the founding fathers). No one has ever seriously doubted that philosophical concepts themselves have presuppositions, even material and economic ones. This is no less true of mathematics and science than of philosophy. If, however, the integrity of concepts themselves are to be reduced to something else—either material, economic, linguistic or psychical—then the burden of proof is upon those who make such claims to demonstrate how and why this is the case. Binswanger’s counterexamples make for an excellent starting point toward this endeavor.

Having argued for the importance of philosophy for rational beings in general, the editors set about to address the issue of why philosophy is particularly germane to the enterprise of business. Rand is generally known as an apologist for capitalism. But what precisely is the connection between philosophy in general and particularly ethics and capitalism? Why is it critical that businesspeople be informed about philosophy? These issues are addressed in a number of pieces that often complement each other nicely. Rand’s piece “Wealth is the product of man’s capacity to think”, an excerpt from *Atlas Shrugged*, is a succinct statement of her understanding of the origin and nature of wealth and its connection both to reason and to virtue. In direct contrast to Marx, Rand holds that it is reason, not labor, which is the origin of wealth. The electric generator as well as farming science are adduced as examples. In the times before these technologies were available, enormous labor was expended merely to produce enough wealth to sustain life. Examples could be multiplied. The tremendous wealth which separates modern from primitive societies in the form of abundant food, heated homes, medicine, and even ‘natural’ resources is not the result of mere labor. Its basis resides in the application of human intelligence to nature.

Much that is left out of the above scenario is supplied by Debi Ghate in a very helpful piece, “The Businessmen’s Crucial Role: Material Men of the Mind”. Ghate acknowledges along with Rand that business people are often not responsible for the science behind technology. The steam engine is a classic example. The principle behind the steam engine was known since the time of ancient Greece but it took an industrialist such as James Watt to recognize its potential and to work relentlessly to the point where it could be produced for the mass market.

Ghate’s piece provides us with real insight and much detail behind the obvious fact that the protagonists behind Rand’s novels are all business people. In a free society in which men live not by force but by trade, only the best products prevail in the marketplace. This simple economic fact holds tremendous ethical significance. When the best product sold at the best price prevails in the market place, the lives of consumers are considerably enriched. Moreover, value is never accidental. The demands of the marketplace for the best products sold at the best prices inevitably call forth the best efforts, the highest expressions of creativity, vision, intelligence and drive on the part of businessmen. Business people such as Hank Reardon and Dagny Taggart are heroes in Rand’s novels because they represent a paradigmatic form of human excellence. For Ghate, the thematization of the businessman as hero is essentially a matter of accurate seeing. If human beings thrive only by shaping and improving their environment, then it is high time we recognize and respect the critical role that business people play in the process of human flourishing. Nowhere are Rand’s Aristotelian roots more evident. In paradigmatic Aristotelian fashion, Rand steadfastly refused the Platonic dichotomy between matter and spirit, soul and body. Thus, she saw business not as a mere material pursuit, but as a spiritual endeavor in which the most fundamental aspects of human creativity, intelligence and drive are actualized and expressed in and through material means. The result—goods and services which immeasurably enrich and extend our lives are concrete embodiments of human virtue. Ghate does an admirable good job of showing us precisely why Rand was such an ardent defender of capitalism. In the free enterprise system, our best efforts are exchanged for the best efforts of others. Only completely free *laissez*-*faire* capitalism honors the ethics of virtue, which is at the basis of this exchange. In arguing for free and unfettered capitalism, Rand did not hold that there should be no rules or laws in which economic intercourse takes place. It is a legitimate and necessary function of government, for example, the judicial system, to enforce contracts and to protect innocent citizens from fraud and abuse. When for example unscrupulous entrepreneurs sell products that cause injury or death, they should be held liable. It is not, however, a legitimate function of government to determine economic outcomes or to redistribute wealth. Ghate effectively conveys Rand’s conviction that any suggestion that one’s best efforts should be taken by force is a symptom of a profound moral and ethical confusion. In short, businessmen need philosophy because philosophy illuminates the moral and ethical basis of the free enterprise system. All too naturally, the moral confusion that makes businessmen into pariahs takes a grandiose form—the form of altruism. Accordingly, the remainder of the book is devoted to a moral defense of capitalism against its altruistic opponents. There are two distinct aspects of Rand’s critique of altruism. The first is a critique of altruism per se one that asserts that altruism is not a virtue. The second is a critique of what might be called the pretentions of altruism and involves the observation that some of the most heinous human evils, for example totalitarianism, are often cloaked in altruistic garb. Some of the most enthusiastic admirers of Rand’s work maintain that the enduring aspect of her critique of altruism involves the second aspect, the first being very poorly supported. Rand herself certainly believed that altruism was not a virtue, but what makes *Atlas Shrugged* a perennial classic is its strikingly original contribution to the hermeneutics of suspicion in the form of a sustained and insightful portrayal of how a plethora of human vices are insidiously and inexorably veiled behind an altruistic facade.

In his essay “The Philosophy of Privation: Environmentalism Unveiled”, Peter Schwartz continues this tradition of a critique not of altruism itself but of evils that appear in altruistic form. While the environmental movement assumes the guise of an unimpeachable altruism, Schwartz adduces some powerful evidence of serious harm that it has done. The essay is certainly destined to become the most controversial piece in the book. While Schwartz leaves us with the impression that the environmental movement is an entire sham, a conclusion not supported by the evidence contained in his premises, the evidence he does provide, particularly concerning the harm caused by the environmental movements’ successful campaign to ban DDT worldwide despite the proven efficacy of DDT as a preventive to malaria, is powerful and convincing. Environmentalists certainly need to respond to Schwartz’s arguments.

In summary, *Why businessmen need philosophy* is a serious, well thought out and enormously informative guide to the philosophy of Ayn Rand, a must-read for business people, business ethicists and for students of Ayn Rand in general.

